# Leçons Sur Le Chancre

**Published:** 1858-07

**Authors:** 


ART. II.—*Leçons sur le Chancre.* Professées par le Docteur RICORD; redigées et publiées par ALFRED FOURNIER, Interne de l'Hôpital du Midi. 8vo., pp. 351. Paris: Adrien Delahaye, 1858.

*Lectures on Chancre.* By Dr. RICORD; edited by ALFRED FOURNIER, Resident-Physician of the Midi Hospital. 8vo., pp. 351. Paris, 1858.

WE are no admirers of Mons. Ricord. Of all the writers on Syphilis, whom it has been our lot to examine, he is the most obscure and unsatisfactory. Filled with overweening conceit and dogmatism, he has done more, in our judgment, to embarrass the progress of our knowledge of syphilis, than any man of modern times. His works are without system or arrangement, and so surcharged with flippancy as to render it extremely difficult to separate the little good they contain from the immense amount of chaff and rubbish in which that little good is buried. If he had set out with a determination to be obscure, he could not possibly have succeeded better. His edition of John Hunter was a reputable production, the more so because he was at the time quite a young man, working for position, and he could, therefore, afford to be clear and intelligible; but when he set up for himself, as "Chirurgien de l'Hôpital du Midi," he strove to become a lawgiver in his particular pursuit, to subvert the facts accumulated by his predecessors, and to set by the ears his contemporaries and compeers. In a word, Ricord became inflated, and, as a consequence, he has ever since been floundering about upon the broad ocean of uncertainty, teaching one thing to-day and another thing to-morrow; and yet, strange to say, this same Mons. Ricord is the very man by whom all other men, especially the little ones, who never have any opinion of their own, and whose nervous system is too feeble to harbor one of any kind beyond the passing hour,—this same Mons. Ricord, we say, is the very man by whom these little men swear, and move, and have their being, upon all topics pertaining to the nature and treatment of syphilis. Dispute anything that has been advanced in the writings of the French syphilographer, and you are at once set down by his friends and admirers as an ignoramus and a heretic, as utterly behind and below the times, as a sort of dark-ages man, whom the light of the sun of science never deigns to visit, to warm, and to illuminate. Whatever he says is law and gospel with them; they swear by it, talk by it, practice by it. There is but one syphilographer, say they, but one expounder of chancre and its effects, but one interpreter of nature in syphilis, and that is "Le Chirurgien de l'Hôpital du Midi." All other men who think and act for themselves in the matter, who dare to form opinions, and carry out these opinions in practice, are old fogies, dolts, asses, for whom there is no hope or salvation either in this world or in the world to come.

We solemnly believe that M. Ricord has done an immense amount of harm as a writer on syphilis, by unsettling the views and practice of the less thinking portion of the profession. The work before us affords ample proof of the truth of this assertion. The "notes et pièces justificatives" of the editor, Alfred Fournier, who is a warm and admiring disciple of Ricord, are too explicit upon the subject to admit of doubt or equivocation. The book everywhere furnishes irrefragable evidence that Ricord has again changed his opinions respecting the nature of chancre, and the writer takes special pains to apologize for his master's many inconsistencies in this, as compared with his former productions. The "notes et pièces justificatives" of Fournier occupy not less than one hundred and fifteen pages of the present work, and it is impossible to peruse them without coming to the conclusion that, even yet, with his vast experience, his notions are not to be received with implicit confidence. He now insists upon the existence of two distinct species of chancre, the indurated and non-indurated, whereas, until lately, he taught that there was only one kind, subject to variation in consequence of local and constitutional causes not connected with the poison of the disease. It is remarkable that a practitioner enjoying so many opportunities of observation, should have been so slow in arriving at this conclusion. The Midi Hospital is the largest depot for syphilitic patients in the world, and if he is really a man of sagacity and judgment, as is alleged he is, it is astonishing that he should have remained so long in doubt upon the subject. The observations of a single year in such an establishment would, one would suppose, have been sufficient for a man of ordinary capacity to produce what it took Ricord twenty-five times that period to produce. The very fact that he has been so long in reaching his present conclusion, so diametrically opposed to those previously attained by him, is sufficient to raise a just doubt in regard to the validity of the doctrines he now teaches. The case of the French syphilographer affords a memorable proof of the circumstance, so often remarked and commented upon, that it is not the number of facts which one observes that imparts perfection to our knowledge of disease and its treatment, but the manner in which they are observed. Ricord has literally lived in a syphilitic atmosphere for a quarter of a century, and yet he has just ascertained that there are two distinct species of chancre, notwithstanding these two species of chancre have daily danced before his eyes, and hourly invited his scientific scrutiny. We do not deny a man the right of changing his opinions; but to change them with every little whiff of wind that sweeps along the surface, is utterly unendurable in a would-be reformer and a would-be scientific teacher. We shall impatiently await the arrival of the next steamer from Havre for further changes in the views of this most sagacious syphilographer. Meanwhile we shall present the reader with an analysis of Mons. Fournier's book, which we deem the more necessary, as it is not likely to meet with an English translation.

Defining syphilis as a contagious malady engendered by a virus, and arising from a chancre, M. Ricord very properly maintains, in opposition to the doctrines of Vidal and his followers, that chancre arises from a chancre, and can alone reproduce it, and that constitutional syphilis recognizes no other origin, unless it be hereditary transmission. In confirmation of the law, that chancre is the necessary antecedent of acquired syphilis, M. Fournier analyzed 826 cases of constitutional syphilis admitted into the hospital in 1856. Of these, 759 were of the secondary, and 56 of the tertiary type. In 815 cases the constitutional effects could undoubtedly be ascribed to a primary sore, and in the eleven exceptional cases that could not be traced directly to a chancre, some were due to hereditary origin, and in others chancrous cicatrices and inguinal enlargements denoted the pre-existence of the primary cause.

As mentioned above, M. Ricord has now abandoned the doctrine of there being but one variety of chancre, and teaches that there exists two distinct forms,—the simple, soft or non-infecting, and the indurated or infecting variety. The simple chancre, which never contaminates the system, is by far the most frequent in occurrence, as will be seen from the following statistics:—The whole number of chancres observed by MM. Fournier and Puche, was 10,341. Of these, 8260 were of the simple, and 2081 of the indurated variety; the proportion being as 4 to 1 in favor of the non-infecting sore. In explanation of this interesting fact, M. Ricord gives two reasons, viz.:—

"Simple chancre is the most fruitful source of the chancrous virus; it is the form of ulceration that secretes pus the best endowed with contagious properties, and which for the longest time preserves the power of inoculating. It does not create an immunity against a new contagion from a chancre of the same species, *i.e.*, against its reproduction in the same individual. It is known, on the contrary, that an indurated chancre occurs, as a general rule, but once in the same subject."

M. Ricord, after an experience of twenty-five years, does not hesitate to affirm that there does not exist any safety against the contagion of a soft chancre, and that the attempts of the supporters of syphilization to change his opinion have been futile. In the therapeutical power, therefore, of inoculation from a simple chancre, as a prophylactic agent against a new attack, he is no believer. In support of this, he instances M. Lindmann, who has inoculated himself over twenty-five hundred times, and never with a negative result.

Proceeding now to a novel question, Ricord states that when he held the doctrine of the pathological unity of both varieties of chancre, he taught without distinction that chancre exhibited itself everywhere; but since his recent researches had led him to distinguish between the two species, he had changed his views. Thus, in his investigations he had seen the indurated chancre appear on every part of the body, but he had never observed simple chancre on the head, including under this term the lips, mouth, nasal fossæ, etc. He is not prepared to deny the possibility of such an occurrence; but, inasmuch as there is no well-authenticated case on record, he does still maintain that soft chancre does not occur on this region, and that when a chancre is situated here it is invariably indurated and accompanied by the proper symptoms of constitutional syphilis. In these views he is sustained by his colleagues, MM. Puche and Cullerier, and in fifty-six cases carefully analyzed by A. Fournier, all the chancres, without an exception, were of the indurated form.

In regard to the mode of propagation and the conditions necessary for the development of the primitive sore, the pus must come in direct contact with the tissues, which must themselves be in a proper condition to receive the influence of the contagious matter. The most favorable state for this action is the presence of an abrasion or slight wound. If the matter be allowed to remain upon a part covered with unbroken epithelium, it will almost certainly produce no result. It is on this account that we are able to handle chancres without becoming inoculated. Not only is this true of the skin, but also of the sound mucous membrane, so that, as has been shown by M. Cullerier and others, a woman who has had connection with a man affected with chancre can inoculate another person, though she herself will remain intact. This is the doctrine of mediate contagion, which has been questioned by some authors and totally disbelieved by others, but which certainly seems to be a settled fact, from several cases adduced in the work before us. As to the virus, it is not necessary that it shall be recent, provided it has undergone no changes which can destroy its contagious property. When a chancre has become gangrenous, it has been proved by inoculation that its pus is harmless.

Inoculation, or artificial contagion, furnishes us a valuable mode of studying the characters of a chancre in nowise different from the ordinary one. This is effected by making a slight puncture in the epidermis with a lancet charged with the virulent matter. The phenomena begin almost immediately, and are as follows:—

"The puncture commences to redden and to be surrounded by a slight inflammatory border, and while this is enlarging it tumefies, and soon a small papule is formed. Upon the top of this the epidermis rises up, and a vesicle filled with serum appears; this enlarges, the fluid grows thick and purulent, and a true pustule succeeds it. This becomes umbilicated at its centre, and after the lapse of a certain time it flattens. Then, one of two events follows: either the pustule breaks, leaving an open ulcer of uniform extent, which constitutes the true chancre, or, the pustule remains intact, dessicates, and is covered by a brownish crust, which, if removed, will expose the specific ulcer, having the following characters:—its circumference is very regularly rounded, provided, always the pustule was developed on a homogeneous tissue; its edges are clearly defined, as if the loss of substance had been produced by a punch; they are, for the most part, a little turned outward and slightly abrupt; under the magnifying glass the edges are slightly jagged. The bottom of the ulcer, most often irregular, as if worm-eaten, presents in general a slightly grayish discoloration; it appears to be covered by a lardaceous substance, a sort of a pseudo-membrane, so adherent that it cannot be detached by washing. The base upon which the ulcer rests is thickened and engorged; it is encircled by an areola of a reddish or violet color, more or less extended outward from the circumference of the ulcer. The secretion is sufficiently analogous in its external characters to the secretion of ordinary wounds; but it is a specific pus on account of the virus it holds in solution."

In the examination the character of the base of the chancre is the most important point to be taken into consideration. The diagnosis is easily established, if there is no inflammatory engorgement, and if it offers to the touch a pliability almost the same as in the surrounding parts. It may, however, happen that the base of the ulcer is thickened to a greater or less degree, imparting to the finger the same sensation as when the base of an engorged boil is pressed. This hardness of the simple chancre is a very different thing from the induration of an infecting chancre, and as such should be carefully examined, so that we may be able to state to the patient with more certainty the probability of his becoming the subject of a loathsome taint of the system; and not only this, for upon the induration depends the course of treatment to be pursued.

Simple chancre may or may not be followed by bubo; thus, of 207 patients affected, 65 had a bubo and 142 had none. Should this accident occur, a remarkable fact, and one known to Hunter, is that it limits itself to the superficial group of lymphatic glands, and that the characteristic primary syphilitic bubo is never met with in the deeper set of ganglions. Even when a superficial gland has become a specific suppurative depot, the pus cannot be transmitted through the inter-lymphatic vessels from one gland to another.

The bubo of simple chancre is highly inflammatory, is painful from the commencement, runs its course rapidly to suppuration, and the pus is inoculable; it is, in fact, but a glandular chancre. Inasmuch as this generally affects but one of the glands in the region in which it occurs, M. Ricord has long since proposed for it the name of *acute monadenitis*.

Simple chancre, which is at the same time the cause of a common irritation and a source of virulence, presents two varieties of bubo. If the chancre acts upon the ganglion merely as a common irritant, it gives rise to a simple adenitis, which is characterized in its progress by the ordinary symptoms of a non-specific bubonocele. It is a ganglionic inflammation which follows the course of other phlegmasias, and may terminate in resolution or in the formation of pus having no specific qualities. The second variety is the acute monadenitis, as described above, of which the pus, when inoculated, gives rise to the pustule of a chancre, and when opened, the wound is itself directly converted into a specific sore.

In explanation of the phenomena that cause the specific character of ganglionic pus, M. Ricord cites the following reason :—

"The virulent pus which bathes the surface of the primitive ulcer penetrates the ulcerated and open extremities of the lymphatic vessels ; this pus rapidly traverses these canals, which it generally leaves unharmed, probably on account of the rapidity with which it traverses them ; then it arrives at the glands, and, being retained in their interior, exercises its specific action, that is, produces a true inoculation, soon followed by the formation of a chancre."

Such is the specific variety of adenitis, to which has been given the name of *bubo by absorption.* Whilst the appearance of the adenitis is almost immediate, and coincides generally with the epoch of the induration of the chancre, in the case of simple chancre there is no fixed time for the lymphatic involvement. It does not show itself always, as does the indurated bubo, in the course of the first or second week, but may appear at a time far distant from the primary sore. Thus, in a case observed by M. Puche, a virulent adenitis showed itself, after the lapse of three years from the time of the appearance of a soft chancre of the serpiginous type. To return again to the simple chancre, which presents in the highest degree specific virulence, a number rather than one will be seen upon the same subject. Thus, of 254 patients presenting simple chancre, only 48 had one, while 206 had a number of chancres, and of this number more than one-half had from three to six. It is this form of chancre which continues the longest time, and preserves its specific qualities almost up to the last moments of its existence. Its tendencies are to destruction, and it generally spreads over a larger surface than the indurated chancre ; in other words, it is the most liable to phagedenic action, which commits such fearful ravages, destroying organs and tissues to a horrible extent. From the preceding statement, M. Ricord, therefore, lays down the following conclusions :—

"Simple chancre is one whose base remains soft or presents merely an inflammatory thickening. It does not react upon the lymphatic ganglions at all, or influences them in a peculiar manner, by producing an acute inflammatory monadenitis, terminating almost always in suppuration, and furnishing most often an inoculable pus. Its edges are clearly defined, and its bottom is irregular and worm-eaten. Most commonly it is at first multiple, or becomes so after a time by a series of inoculations in its neighborhood. Its pus is, *par excellence*, virulent and contagious, preserving for a long time its specific qualities. Finally, this form of chancre has a destructive and invading tendency, and is most apt to undergo the phagedenic variation."

The true pathognomonic character of chancre is that its pus is capable of producing, by inoculation, an ulcer similar to that by which it has been furnished.

In regard to the prognosis of chancre, M. Ricord, with the authority of long experience, insists that "simple chancre, with a soft base, is a purely local affection, which limits its effects to the region which it attacks and is never followed by constitutional accidents. In other words, it is a chancre which does not infect the system,—a chancre without syphilis." If this be true, how important is it that the diagnosis should be made out upon the first examination, so that the practitioner and patient shall know with certainty what the consequences shall be.

Regarding simple chancre, then, as a mere local affection, M. Ricord directs his treatment to it alone; the great object being to reduce the specific ulcer to the state of a simple sore,—to destroy the chancre as soon as possible. This is to be effected by a deep, destructive cauterization; and after successively experimenting with Vienna paste, caustic potash, &c., he now proposes a new agent of "*wonderful efficacy*," but whose application is very painful. This caustic is composed of sulphuric acid and powdered vegetable charcoal united in the proper proportions to form a semi-solid paste. This is to be spread upon the whole surface of the chancre and a slight distance beyond it; it soon dries, and forms a black adherent crust, which generally drops off, in the course of the second week, leaving a healthy ulcer, in every respect analogous to that which follows the separation of an eschar. Sometimes, indeed, the reparative process is almost finished, and occasionally a cicatrice is formed by the time the paste has come away. Should, however, the patient refuse to submit to cauterization, the most simple hygienic means will furnish the best results. Thus, if the chancre has no tendency to spread, emollient or slightly astringent lotions, frequently renewed, and followed by the application of dry lint to the ulcer, will answer a good purpose. If the suppuration be abundant and the chancre is extending, aromatic wine will be found to be one of the very best topical remedies. The patient is to wash the affected part three or four times a day with this, and then cover it with a piece of fine lint merely moistened with the wine. Eight or ten grains of the gummy extract of opium should be added to the ounce of aromatic wine, if the chancre be accompanied with much irritability and pain, and opium should also be administered internally. When the chancre takes on phagedenic action, M. Ricord has the greatest confidence in the tartrate of iron and potassa, which seems to exert some specific influence upon it; and although he looks upon cauterization as an excellent preventive and curative agent of phagedena, yet he has combated the greater number of these cases with the above remedy. His formula is one ounce of tartrate of iron and potassa to about six and a half ounces of distilled water. Of this, a tablespoonful is to be taken three times a day, and the ulcers are to be washed and dressed with the same liquid twice in the twenty-four hours. On two occasions M. Ricord saw enormous phagedenic chancres, which had resisted every form of treatment, modified and cured by an intercurrent erysipelas. Nothing can be more hurtful to a simple chancre than fatty substances; and, above all, mercurial ointment should be scrupulously withheld, for it is in itself one of the most frequent incentives to gangrenous action. The ordinary treatment is applicable in larvated chancre when seated just behind the meatus, observing the precaution to keep the lips of the orifice separated by a small cone of lint. When it is seated farther back in the urethra, and is attended with very acute symptoms, antiphlogistic treatment is indicated; fifteen or twenty leeches to the perinæum or pubes, baths and copious drinks will be useful. Above all things, morbid erections, which by distending the affected part cause rents, and a further extension of the chancre, must be avoided by appropriate remedies. When the acute symptoms have subsided, injections, several times daily, of equal parts of aromatic wine and decoction of poppy heads, will be found to be a very simple and good mode of medication If this does not produce irritation, the pure wine may be substituted. Chancres seated far back in the vagina and on the neck of the uterus should be treated on the same principles as when the chancre is external.

M. Ricord very properly condemns mercury, during the course of a simple chancre, as favoring the development of phagedenic action. No constitutional treatment at all, as far as the affection itself is concerned, is required; should the patient be weakened by privation and disease, tonics and sustaining measures are indicated; if, on the contrary, he be robust and plethoric, a slightly debilitating regimen will do good.

Entering now upon the consideration of infecting or indurated chancre, that which alone is capable of producing a constitutional affection, the first noticeable fact is that it is natural to man alone; for it is well established, that of the innumerable attempts that have been made to inoculate animals, not a single one has furnished the specific pustule. This form of chancre, unlike the soft variety, can be developed on any part of the body, from the crown of the head to the sole of the foot; nor are the mucous membranes any more exempt from it than is the skin. It generally developes itself without pain, and in so slow and insidious a manner as that it is not uncommon to see persons affected with a large sore of which they had not the slightest suspicion.

The form of an indurated chancre in nowise differs from that of a simple one; sometimes it is a pustule, at others, a true chancre from its commencement. To an experienced eye there are, however, some slight differences. The surface is smoother, presenting a less worm-eaten and jagged appearance; the bottom is of a uniform grayish tint; the borders are generally smooth and shining, as if varnished, and insensibly slope toward the bottom of the ulcer, which has a cupelliform appearance. It is most often single, small in extent, and rarely becomes phagedenic. It suppurates but slightly, the discharge being of a saneous, serous nature. The great pathognomonic sign is the induration which surrounds and lies under the ulcer. This is due to effusion of lymph in the absorbent vessels and in the adjacent cellular tissue, and imparts to the touch an elastic cartilaginous sensation which cannot be mistaken. Bell likens it to the hardness of the section of a dry pea, and this is the most frequent form of induration. There is one variety characterized by its being superficial, merely lining the base of the ulcer, which, when pressed, imparts to the touch a sensation like that produced by lightly pressing with the fingers the opposite sides of a piece of parchment. To this, M. Ricord has given the name of *parchment induration,* and it is sometimes so delicate and superficial as to require some tact to discover it. In regard to the time of the appearance of the induration, M. Ricord states he has never met with it before the third day, and positively denies that it does in any case precede the ulcer, which is the ground held by G. Babington. It begins to show itself generally toward the end of the first week, and becomes more marked in the second week after the infecting coitus. The induration is most evident in those tissues which abound in lymphatic vessels, as for example, the frænum and lips of the meatus; and, inasmuch as they do not exist in such numbers in the mucus membrane of the vagina, in the carunculæ myrtiformes, the anus, &c., it is not so well marked. In these cases it is of the parchment variety, which is not only difficult of detection while it actually exists, but which often disappears before the process of cicatrization has been completed. Hence it is that many physicians have concluded that an infecting chancre in females cannot be indurated, attributing the constitutional effects to a simple chancre. They must take the above facts into consideration, and remember that an experienced touch is necessary to detect the slightly indurated sore. M. Ricord, therefore, insists that induration is always the prelude to constitutional syphilis in women.

Caustics have the power of hardening the tissues and creating an artificial indurated base in chancres which have been subjected to their action. Such is the effect of nitrate of silver, the mineral acids, alum, tannin, and other articles; but more especially is this true of corrosive sublimate and chromate of potassa, which indurate the parts in such a manner as to deceive the most practiced touch. In case of doubt, however, another sign of the greatest diagnostic value is the indurated, indolent bubo, which is produced exclusively by the infecting chancre, and invariably accompanies it. Its appearance, generally, coincides with the period of the induration of the chancre, and almost always shows itself during the first or second week. It is multiple, attacking several glands simultaneously, which impart to the touch the same feeling as does the base of the indurated sore. It is not painful, is very indolent, remaining a long while after the cicatrization of the primary sore, and evinces no disposition to become inflamed. Except from an outside irritation, this form of bubo never suppurates; that is, specifically, as is proved by the fact that the pus when inoculated is incapable of producing the characteristic pustule of chancre.

M. Ricord has long since maintained that when induration exists it is necessarily followed by constitutional symptoms, that it is, in fact, less a cause than an effect, being but the first of the secondary accidents. The taint of the system, if the sore be left alone, will appear before the lapse of six months. He divides syphilis, as formerly, into three stages; the first being marked by chancre and bubo, the second by affections of the superficial tissues, and the third by affections of the deep tissues. As regards the contagiousness of each, the primary is inoculable by actual contact; the secondary is not, but is transmissible from parent to offspring; the tertiary being neither transmissible nor inoculable, but the children of parents so affected are susceptible to scrofulous disease.

The manner in which the system becomes infected by the syphilitic virus, how it is transmitted to the different organs and through the different tissues, is not certainly known. It is justly supposed that it is effected through the medium of the blood; but this is not found to contain any contagious property, though it is modified, as the examination of the blood of persons affected with an indurated sore shows an increase in the albumen and a diminution of the globules. We look in vain for the cause in the different secretions, nor does even the pus of any of the secondary or tertiary accidents possess any inoculable property on healthy persons. This is effectually proved by the experiments of MM. Cullerier, Sarrhos, Fournier, and others, upon their own persons. Inoculation, too, of the morbid products of secondary or tertiary syphilis upon syphilitic subjects is always sterile; thus proving the Hunterian doctrine "that the constitutional symptoms of syphilis do not produce a pus similar to that from which they arise."

In the same way that vaccination affords immunity from an attack of smallpox, so does indurated chancre produce a diathesis, which prevents the system from being a second time affected. As a law, M. Ricord lays down that indurated chancre occurs but once in the same subject. This is substantiated by the fact, that when a syphilitic person is inoculated with the pus of an infecting chancre, either no result at all follows, or a simple, soft chancre is produced, which throughout its whole course does not exhibit a single sign of induration. This simple sore will, however, produce in healthy persons a true indurated sore, thus showing that syphilis can be transmitted by a subject previously affected contracting a new chancre From the most recent researches on the contagion of chancre, M. Ricord, therefore, teaches that simple chancre in uncontaminated persons reproduces a simple chancre. Indurated chancre, under the same circumstances, reproduces its own special variety. Indurated chancre is transmitted to subjects previously syphilitic under the form of a simple chancre, which, in its turn, may be transmitted either as an infecting or a non-infecting sore.

In the treatment of this form of chancre, it is of the utmost importance that it should be attended to in its early stages before induration has appeared; for then it is but a local affection. By its complete destruction, converting it into a simple sore, and depriving it of all infectious properties, the systemic taint may be warded off. This may be effected by excision when practicable, or with equal certainty by the carbo-sulphuric paste. M. Ricord states that of all the cases which he has seen cauterized, within the first four days, not a single one has been followed by constitutional symptoms. Should the patient present himself at a later period, or should the sore have been neglected, this procedure will be useless; the indication then being to treat the diathesis. The specific for incipient constitutional syphilis, both as a preventive and curative agent, is mercury, and the form preferred by M. Ricord is the protiodide. He administers this so soon as the induration appears, and condemns those physicians who wait for more painful and troublesome symptoms before beginning this course. Although the treatment is generally so beneficial in its results, it is not to be indiscriminately employed; for, when the slightest doubt exists in regard to the nature of the chancre, we must wait and withhold mercury. If the diathesis exist, it will not fail to show itself in the course of a few weeks; but if no symptoms appear by the sixth month, we may assure the patient of his present and future safety.

This course of treatment should not be carried to salivation; for then its curative action is generally suspended. Should it, however, supervene, it can be combated by the use of chlorate of potassa.

In a note concerning this remedy, we find that M. Fournier has come to the following conclusions:—

"A mercurial stomatitis being produced, it is not necessary to suppress the specific medication to cause the accident to disappear. Chlorate of potassa, employed in conjunction with the mercury, suffices for its cure. In the progress of a stomatitis, the dose of the mercurial preparation can even be increased, jointly with the administration of the chlorate, in those cases in which the gravity of the syphilitic symptoms calls for the immediate intervention of an energetic medication. Chlorate of potassa is not only a curative agent in ptyalism, but can be employed equally well as a prophylactic."

As mercury is the specific for secondary, so is the iodide of potassium the specific for tertiary syphilis. Inasmuch as it is not only curative, but also preventive, it should always succeed a mercurial course, in order to prevent entirely or retard tertiary symptoms. After attentive and patient observation as to the curability of syphilis, M. Ricord has concluded that the best results are afforded by placing the patient on a six months' course of mercury, after which time the iodide of potassium is to be administered for three months. This plan, "in the great majority of cases, succeeds in neutralizing the poisonous virus."

Having thus considered, in detail, both varieties of chancre, M. Ricord, in his last lecture, gives the following *résumé* of the characteristic points of each:—

| SIMPLE CHANCRE. | INFECTING CHANCRE. |
|---|---|
| 1. Is the most common form; its base is soft; is generally multiple, and may be so from the commencement. | 1. Is met with less frequently; the base is indurated; is generally single; rarely multiple. |
| 2. The pus possesses the most contagious properties, and is inoculable during almost the whole time of the existence of the chancre. | 2. The pus soon loses all specific virulency, at least on the infected subject, who becomes, in a few days, refractory to inoculation from his own virus. |
| 3. Its tendencies are to spread and destroy. It is the variety most apt to undergo phagedenic action. | 3. Shows little disposition to enlarge, soon limits itself, and spontaneously heals. Rarely becomes phagedenic. |
| 4. It does not occur on the cephalic region. | 4. Appears on every part of the body. |
| 5. Is probably transmissible to animals, and can be reproduced, *ad infinitum*, upon the same individual. | 5. Affects man alone, and can appear but once under its own proper form. |

6. Bubo is not a necessary consequence; but when it does occur it is acute, mono-ganglionary, and commonly suppurates, the pus being inoculable.

6. Invariably followed by lymphatic involvement. The bubo is chronic, indurated, and affects several glands. Rarely suppurates, and the pus is not specific.

7. Its origin is a simple chancre, which reproduces its own variety.

7. Its origin is an indurated chancre, which equally reproduces its own variety.

8. It is merely a local affection, producing no influence upon the system.

8. Creates a diathesis, and is the beginning of constitutional syphilis.

Although M. Ricord insists on the unity of the syphilitic virus and two varieties of chancre, he is not prepared to say whether or not there are two varieties of chancrous virus. Even when it has been shown that these two forms of chancre belong to two distinct pathological varieties, it does not invalidate the fact of there being but one syphilitic virus; but merely demonstrates that there is, besides syphilis, an affection manifesting itself by a chancre, having no infectious influence upon the economy. From this, the legitimate conclusion would be, that there are two viruses, one belonging to syphilis, and producing an infecting chancre, the other being a chancrous virus independent of syphilis. Still, the question in regard to the duality of the chancrous virus is an hypothesis which time will have to clear up; while the unity of the syphilitic virus seems to be a well-established fact.

We have now finished our task of presenting to our readers an analysis of these lectures. Without entering into any criticisms upon their merits, we rather prefer to bring them forward, and leave each one to judge of them for himself. The work contains needless repetition, a fact probably owing to M. Ricord's desire to impress its contents more fully upon his readers. M. Fournier has acquitted himself creditably in arranging these lectures, and has taken considerable pains with the additions which he has made to the work.

